# Ultra-Solvatochromic Probe Based on Binaphthofuran for Visual Detection of Early Trace Oil Leakage in Oil Reservoir Capsules

**DOI:** 10.3390/molecules31193459

**Published:** 2026-09-28

**Authors:** Yihang Zhang, Hanlin Wen, Fuzhi Li, Xinyuan Huang, Wenqi Fu, Shanshan Yu

**Affiliations:** 1 State Grid Sichuan Electric Power Company DC Branch, Chengdu 610021, China; 2 Sichuan Houying Electric Power Technology Service Co., Ltd., Deyang 618000, China

**Keywords:** solvatochromic probe, fluorescent visual detection, binaphthofuran, early trace oil leakage detection, oil reservoir capsules

## Abstract

The long-term reliability of converter transformers hinges on the integrity of oil reservoir capsules, whose early-stage oil leakage detection remains challenging. We present Probe (*R*)-**1**, a novel D–π–A solvatochromic fluorescent probe synthesized via a binaphthofuran donor–π-bridge scaffold functionalized with a *p*-trifluoromethylbenzoyl acceptor, designed for rapid, visual, on-site identification of trace oil leaks. The probe exhibits a pronounced positive solvatochromic shift of 138 nm across solvent polarity gradients, enabling unambiguous, naked-eye discrimination of structurally similar organic liquids through distinct fluorescence color transitions. In ethanol solutions, the probe achieves an exceptionally low detection limit of 5.88 μL/mL for insulating oil, surpassing industry sensitivity requirements. On real-world oil capsule surfaces, the probe produces a stark fluorescence contrast between the oil stains and the capsule background, permitting clear visualization of micro-volume droplets under a 365 nm handheld UV lamp. This method eliminates reliance on complex instrumentation, offering a rapid, cost-effective, and field-deployable solution for proactive maintenance in power grid infrastructure.

## 1. Introduction

Oil leakage from transformer reservoir capsules is a long-standing hidden hazard in substation operation, and early identification of trace leakage can effectively prevent subsequent insulation degradation and power accidents [1,2,3]. Traditional detection methods, such as pressure testing, X-ray imaging, and infrared thermography, are limited by expensive equipment, complex operation, and low sensitivity to early-stage micro-leaks [4,5,6,7,8]. Although a fluorescence-based oil leak detection strategy has been reported [9], it relies on the intrinsic fluorescence of transformer oil and suffers from two critical limitations: (i) its signal-to-noise ratio is inherently low due to strong interference from background fluorophores such as surface dust and aged rubber debris on the inner capsule surface; (ii) its emission lies in the ultraviolet (UV) region, requiring benchtop instrumentation rather than simple visual observation. To the best of our knowledge, no rationally designed fluorescent molecular probe has been reported for transformer oil leakage detection on capsule surfaces. Therefore, herein, we present a fundamentally different strategy based on a molecular solvatochromic probe that transduces the polarity difference between low-polarity mineral insulating oil and high-polarity rubber matrix/background contaminants into a distinct dual-color fluorescence contrast, enabling high-contrast naked-eye detection of trace oil leakage under a simple handheld UV lamp.

In recent years, fluorescent probes have attracted widespread attention in trace substance sensing due to their high sensitivity, low detection limit, rapid response, and capability for visual identification without reliance on large-scale instrumentation [10,11,12,13,14,15]. Solvatochromic fluorescent probes, operating mainly via intramolecular charge transfer (ICT) or excited-state intramolecular proton transfer (ESIPT) mechanisms, exhibit significant shifts in fluorescence emission wavelength in response to changes in microenvironmental polarity or hydrogen-bonding strength, enabling environment-responsive optical signaling and ratiometric detection with inherent anti-interference capability [16,17,18]. These probes have been successfully applied to solvent polarity analysis, food quality assessment, and biological imaging with high selectivity [19,20,21]. However, their application in industrial equipment inspection, particularly for on-site visual detection of trace oil leakage in power systems, remains largely unexplored.

1,1′-Bi-2-naphthol (BINOL)-based fluorescent probes have been extensively developed for chiral recognition of amino acids, small organic molecules and metal ions over the past decades [22,23,24,25,26]. Compared to regular BINOL derivatives, the binaphthofuran scaffold provides a large, rigid π-system, enabling strong fluorescence with high quantum yields in the visible region. The furan ring enhances conjugation between the donor and the acceptor groups, promoting efficient ICT that is the basis of the solvatochromic response. Also, BINOL derivatives can be readily functionalized at the 3,3′-positions through well-established chemistry, allowing facile tuning of the donor–acceptor properties. It is worth noting that the parent binaphthofuran scaffold has been previously reported as an aggregation-induced emission (AIE)-active luminogen with high fluorescence quantum yield and large Stokes shift for cell imaging applications [27].

Herein, a D–π–A fluorescent probe was synthesized by direct conjugation of a furan unit with a 1,1′-bi-2-naphthol (BINOL) scaffold, exhibiting a solvatochromic emission shift of over 138 nm from deep blue to orange-red as solvent polarity increased. This probe demonstrates distinct fluorescence color contrast between low-polarity insulating oil phase and high-polarity oil reservoir capsule background. Only a low-cost handheld UV device is needed to clearly identify the tiny oil stain areas on the capsule surface, thus facilitating on-site oil leakage inspection. The detection limit of insulating oil in ethanol solution reaches 5.88 μL/mL, which fully meets the sensitivity requirements of the power industry for early oil leakage detection, offering a practical, cost-effective technical path for proactive fault detection in power grid maintenance workflows.

## 2. Results and Discussions

### 2.1. Probe Design and Synthesis

To achieve on-site visual detection of early oil leakage in transformer oil reservoir capsules, this study designed and synthesized a D–π–A type solvatochromic fluorescent probe (*R*)-**1** based on a binaphthofuran skeleton. Leveraging the intrinsic electron richness of the binaphthofuran skeleton as the donor, we introduce a *p*-trifluoromethylbenzoyl moiety at the 3-position of the furan ring as the electron-withdrawing end, where the carbonyl group serves as the primary acceptor site and the CF_3_ group further amplifies the pull–push electronic effect across the conjugated system. This deliberate donor–acceptor arrangement renders the molecule a substantially larger dipole moment in the excited state than in the ground state, a hallmark of strong ICT character, so that fluorescence emission undergoes a pronounced red shift with increasing solvent polarity, providing a clear colorimetric readout of environmental polarity changes. Whereas the parent binaphthofuran was previously explored as an AIE-active bioprobe, the present study repurposes the scaffold into a polarity-sensitive solvatochromic sensor via the trifluoromethyl benzoyl functionalization, demonstrating a new application of this fluorophore class in industrial power-equipment fault diagnosis.

The material of transformer oil pillow capsules is usually nitrile rubber or acrylate rubber, which has relatively high polarity, while transformer oil is mainly composed of highly refined mineral hydrocarbons, with alkanes, cycloalkanes and aromatic hydrocarbons as its core components, and it is very low in polarity. It is expected that this probe can form a visually distinguishable fluorescence emission contrast at the interface between the low-polarity insulating oil and the high-polarity rubber background.

The fluorescent probe (*R*)-**1** was prepared from (*R*)-BINOL-derived dialdehyde and excess 2-bromo-4′-(trifluoromethyl) acetophenone under alkaline conditions with K_2_CO_3_ (Scheme 1). The crude product was purified by silica gel column chromatography and recrystallized from ethyl acetate (EA) to obtain a yellow needle-like solid, with a yield of 59%. ^1^H NMR, ^13^C NMR, ^19^F NMR, and HRMS characterizations confirmed the accurate structure of the probe.

The solvatochromic fluorescence properties of probe (*R*)-**1** originate entirely from its D–π–A electronic structure and ICT behavior, which are completely independent of the molecule’s absolute configuration. As a result, a racemic mixture of probe **1** exhibits identical photophysical performance, including the same absorption and emission wavelengths, fluorescence quantum yields, and solvatochromic response profiles, as the enantiopure (*R*)-form used in this work. This feature provides an obvious practical advantage for subsequent industrial translation: racemic BINOL, which is significantly lower in cost than enantiopure BINOL, can be fully adopted as the starting material for large-scale probe production, without any compromise to the sensing performance for mineral insulating oil leakage detection.

### 2.2. Solvatochromic Properties of Probe (R)-***1***

To study the solvatochromic properties of probe (*R*)-**1**, we dissolved probe (*R*)-**1** in 14 organic solvents, including toluene (TOL), dichloromethane (DCM), chloroform (CHCl_3_), carbon tetrachloride (CCl_4_), n-hexane (HEX), diethyl ether (Et_2_O), tetrahydrofuran (THF), dioxane (DIO), ethyl acetate (EA), N,N-dimethylformamide (DMF), dimethyl sulfoxide (DMSO), methanol (MeOH), ethanol (EtOH) and isopropanol (iPrOH), with the probe concentration at 2.0 × 10^−5^ mol/L for all solutions. We tested the fluorescence emission spectra and ultraviolet absorption spectra at room temperature.

From the image under 365 nm UV lamp irradiation in Figure 1, it is observed that probe (*R*)-**1** has obvious solvatochromic properties, showing a rich variety of colors in solvents of different polarities, with the color range spanning from deep blue to orange-red. As shown in Figure 2a and Table 1, as the solvent polarity increases, the fluorescence emission continuously red-shifts, moving from 456 nm in n-hexane solution to 594 nm in methanol solution, with the red-shifted wavelength as high as 138 nm. This phenomenon where fluorescence red-shifts with the increase in solvent polarity is called positive solvatochromism [28]. In addition, as the solvent polarity increases, the emission peak also keeps widening, which is a typical characteristic of fluorescence caused by ICT mechanism.

Different from the fluorescence spectra, the ultraviolet (UV) absorption spectra are barely affected by these solvents. As shown in Figure 2b, the ultraviolet absorption curves of probe (*R*)-**1** in all solvents show maximum absorption at around 344 nm and a shoulder peak at around 392 nm. The influence of solvents on the fluorescence emission spectra is far greater than that on the UV absorption spectra, which indicates that the dipole moment of the molecule in the excited state is larger than that in the ground state [29]. The Stokes shift also increases significantly with the increase in solvent polarity, ranging from 112 nm in n-hexane to 254 nm in MeOH, which is generally attributed to the reduction in excited state energy caused by solvent dipole relaxation [30].

To further discuss the relationship between its solvatochromism and solvent polarity, we referred to E_T_(30) [31], an empirical parameter commonly used to describe solvent polarity. As shown in Figure 3, we performed linear fitting with the maximum emission wavelength (nm) against E_T_(30), and the obtained linear correlation coefficient R^2^ is 0.98, indicating a strong overall linear relationship. This shows that the fluorescence emission peak based on intramolecular charge transfer characteristics has extremely strong dependence on solvent polarity. We note that protic solvents (methanol, ethanol, isopropanol) show slight positive deviations from the linear fit compared to aprotic solvents, which can be attributed to hydrogen-bonding interactions that provide additional stabilization of the ICT excited state beyond pure dipolar interactions.

Based on the excellent solvatochromic effect of probe (*R*)-**1**, we tried to use it for efficient visual distinction of organic liquids with similar structures. We selected three groups of solvents with similar structures: halogenated hydrocarbons with the same halogen element (DCM, CHCl_3_, CCl_4_), ethers (Et_2_O, THF, DIO), and alcohols (MeOH, EtOH, iPrOH). As shown in Figure 4, for all these three groups of probe (*R*)-**1** solutions with similar structures, obvious differences were observed in the fluorescence under 365 nm handheld UV lamp irradiation, with a high degree of distinction. In the halogenated hydrocarbon group, the DCM solution emits yellow-green fluorescence, CHCl_3_ emits green fluorescence, and CCl_4_ solution emits blue fluorescence. In the ether group, Et_2_O shows bright blue fluorescence, THF shows yellow-green fluorescence, and DIO shows blue-green fluorescence. In the alcohol group, MeOH shows orange-red fluorescence, EtOH shows orange fluorescence, and iPrOH shows yellow fluorescence. This method of identifying organic solvents is simple, fast, and has excellent visualization effect, so it has significant potential in the rapid distinction of similar-structured organic liquids.

### 2.3. Visual Monitoring of Early Trace Oil Leakage in Oil Reservoir Capsules

Transformer oil is a typical mineral insulating oil (Kunlun KI50X) composed primarily of alkanes (~60%), cycloalkanes (~30%), and aromatic hydrocarbons (~10%), with trace amounts of sulfur compounds and antioxidant additives. Its dielectric constant is approximately 2.2, consistent with a low-polarity non-polar solvent.

Leveraging the probe’s exceptional solvatochromic discrimination capability, we aim to apply probe (*R*)-**1** for the detection of oil leakage in oil reservoir capsules. To evaluate the detection sensitivity of probe **1** toward insulating oil, we first conducted experiments in ethanol solution, chosen for its practicality and environmental compatibility in routine applications. The concentration of probe (*R*)-**1** in ethanol was fixed at 2.0 × 10^−5^ mol/L, and fluorescence spectra were recorded across a range of insulating oil concentrations from 1 to 100 μL/mL in EtOH. All measurements were performed in triplicate, and the reported values represent the average of three independent replicates.

As shown in Figure 5a, the maximum emission wavelength of probe (*R*)-**1** in EtOH is initially at 585 nm. Upon the addition of insulating oil, a progressive blue shift is observed, reaching 569 nm at a concentration of 100 μL/mL; concurrently, the fluorescence intensity increases with rising oil content. The limit of detection (LOD) was calculated using the formula LOD = 3σ/S, where σ is the standard deviation of fluorescence intensity from ten blank samples (σ = 15.6812), and S is the slope of the linear calibration curve between fluorescence intensity at 424 nm and oil concentration (S = 7.99784), yielding an LOD of 5.88 μL/mL. As shown in Figure 5b, a strong linear relationship exists between fluorescence intensity and oil concentration over the range of 1–100 μL/mL, with a correlation coefficient R^2^ = 0.99585. This result demonstrates that probe (*R*)-**1** exhibits high sensitivity toward insulating oil, meeting the requirements for early detection of trace oil leakage and providing a reliable quantitative basis for subsequent real-sample analysis.

To evaluate the visual detection ability of probe (*R*)-**1** for oil leakage on oil reservoir capsules, this study selected three commercial capsule brands—A, B, and C—as experimental samples, systematically optimizing the probe concentration. Using ethanol as the solvent, four concentration gradients of the probe solution were prepared: 5.0 × 10^−4^ mol/L, 2.5 × 10^−4^ mol/L, 1.0 × 10^−4^ mol/L and 5.0 × 10^−5^ mol/L. In each experiment, 10 μL of insulating oil was first deposited onto the capsule surface, followed by the addition of 50 μL of the probe solution, with fluorescence response observed under 365 nm UV light. As shown in Figure 6a–c, oil stains without probe addition exhibited no fluorescence emission. In contrast, upon addition of the probe (Figure 6d–f), the distinct solvatochromic behavior of probe molecules in the high-polarity ethanol phase versus the low-polarity oil phase generates a clear fluorescence color contrast between the oil stain and the surrounding solvent background, enabling visualization of leakage locations. Optimization results indicate that at excessively high probe concentrations (e.g., 5.0 × 10^−4^ mol/L, 2.5 × 10^−4^ mol/L), the fluorescence intensity was overly strong, diminishing the brightness differential between oil and ethanol regions. At excessively low concentrations (e.g., 5.0 × 10^−5^ mol/L), the signal is too weak for reliable naked-eye detection. At 1.0 × 10^−4^ mol/L, all three capsule samples exhibit a stable, high-contrast dual-color fluorescence pattern: oil stains emit blue-green fluorescence, while the ethanol background displays orange-red fluorescence, yielding the optimal imaging performance. Consequently, 1.0 × 10^−4^ mol/L was selected as the standard working concentration for subsequent visual detection applications.

Based on the optimized probe concentration of 1 × 10^−4^ mol/L, further optimization of the detection method, specifically, the application technique of the probe’s ethanol solution, was conducted by comparing three approaches: full-surface coverage, droplet deposition, and spray application. It should be noted that a typical commercial transformer oil reservoir capsule has an approximate size of 6 m in length and 3 m in diameter, with a total inner surface area of dozens of square meters (Figure 7a). Given such a large surface, trace oil leakage at the early stage usually forms only thin, transparent oil films or tiny spots on the rubber inner surface, which are extremely difficult to identify with the naked eye under natural visible light and can easily be missed during routine manual inspection (Figure 7b,c). As shown in Figure 7d–f, oil without the addition of the probe exhibited no fluorescence emission under 365 nm UV irradiation. With application of the probe solution, results demonstrated that all three methods produced detectable fluorescence responses (Figure 7g–i), of which the spray application method yielded the most superior coloration performance. Under 365 nm UV irradiation, capsules treated with the spray method exhibited a clear fluorescence contrast: oil-contaminated regions emitted bright green fluorescence, while oil-free regions displayed yellow fluorescence, creating a sharp visual distinction. This high-contrast pattern enables accurate and rapid identification of early trace oil leakage on the capsule surface.

Gasoline, kerosene, diesel and other petroleum-derived products all produce a similar blue-green fluorescence response, confirming that the sensing mechanism of this solvatochromic probe is driven by environmental polarity rather than specific chemical recognition of transformer oil molecules. In actual transformer oil reservoir capsule inspection, the only low-polarity petroleum-based substance that may leak and contact the capsule rubber matrix is the internal insulating mineral oil itself, while gasoline, kerosene, diesel and other similar petroleum products do not exist in the normal operation environment of substation equipment. This scenario specificity ensures that the probe can accurately identify the trace oil leakage of the capsule without being interfered with by irrelevant petroleum pollutants.

Unlike tracer methods that require adding foreign substances into the insulating oil (which may affect oil properties and cause serious accidents in substation operation), our approach is completely noninvasive to the oil system. In addition, while commercial fluorescent oil tracers rely mostly on a single-color fluorescence intensity change, our probe provides a ratiometric, dual-color (blue-green vs. orange-red) response based on solvatochromism, offering inherently higher contrast and better anti-interference capability.

## 3. Materials and Methods

### 3.1. Materials

(*R*)-2,2′-dihydroxy-[1,1′-binaphthalene]-3,3′-dicarbaldehyde was synthesized as previously described [32]. All chemicals and reagents were of analytical grade and used without further purification. All solvents for the optical spectroscopic studies were of high-performance liquid chromatography grade. Analytical-grade solvents and all reagents were purchased from Energy Chemical Co., Ltd. (Shanghai, China) and Sigma-Aldrich (Shanghai, China). Oil reservoir capsules were commercially available.

### 3.2. Instruments

Nuclear magnetic resonance: Bruker AV-400 nuclear magnetic resonance spectrometer (^1^H: 400 MHz, ^13^C: 100 MHz, ^19^F: 376MHz), Bruker BioSpin, Fällanden, Switzerland.

Mass spectrometry: SCIEX X500R QTOFMS mass spectrometer, SCIEX, Framingham, MA, USA.

Fluorescence spectrum: F-7000 fluorescence spectrometer, Hitachi High-Tech, Tokyo, Japan.

Absolute fluorescence quantum yield: Horiba JoblnYvon-Edision Fluorolog-3, Horiba Scientific, Edison, NJ, USA.

Ultraviolet spectrum: U-1900, Hitachi High-Tech, Tokyo, Japan.

Optical rotation: IP120 automatic polarimeter, Shanghai Yimai Instrument, Shanghai, China.

### 3.3. Probe Preparation and Characterization

K_2_CO_3_ (2.20 g, 16.0 mmol), (*R*)-2,2′-dihydroxy-[1,1′-binaphthalene]-3,3′-dicarbaldehyde (0.68 g, 2.0 mmol), 2-bromo-4′-(trifluoromethyl) acetophenone (0.91 g, 4.8 mmol) and 30 mL of acetone were added into a 100 mL single-neck flask. The mixture was stirred and refluxed overnight and then cooled to room temperature. The solvent was removed under reduced pressure, and the mixture was extracted with water and ethyl acetate. The water layer was extracted three times with ethyl acetate, and the collected organic layer was washed twice with saturated brine, dried over anhydrous MgSO_4_, and then evaporated under reduced pressure. Purification was performed by silica gel column chromatography (petroleum ether:DCM = 1:1) to obtain a yellow powder, which was then recrystallized from EA to obtain 0.8 g of yellow needle-like solid, with a yield of 59%.

^1^H NMR (400 MHz, Chloroform-d) δ 8.51 (s, 1H), 8.18 (d, *J* = 9.1 Hz, 1H), 7.94 (d, *J* = 8.1 Hz, 2H), 7.87 (s, 1H), 7.59–7.54 (m, 2H), 7.49 (d, *J* = 8.1 Hz, 2H), 7.45–7.40 (m, 1H). ^13^C NMR (101 MHz, Chloroform-d) δ 182.9, 154.1, 152.9, 139.5, 134.4, 134.1, 132.8, 131.6, 130.1, 129.5, 127.3, 127.1, 126.1, 125.4, 125.4, 125.4, 125.3, 125.0, 125.0, 123.9, 122.3, 116.4, 113.0. ^19^F NMR (376 MHz, Chloroform-d) δ −63.20 (s, 6H). HRMS (ESI) *m*/*z*: [M + H]^+^ Calcd for C_40_H_21_F_6_O_4_^+^: 679.1344; Found: 679.1336. [α]_25_^D^ = +28 (c = 10 g/L, CHCl_3_).

### 3.4. Sample Preparation for Detection Limit Measurement

The probe **1** stock solution in ethanol (2.0 × 10^−3^ mol/L) was freshly prepared before each test. To a solution of probe **1** (2.0 × 10^−3^ mol/L) in EtOH (20 μL), the corresponding amount of insulating oil (0–100 μL) was added, and the mixture was diluted to 2 mL with EtOH. All samples were tested within 30 min.

### 3.5. Oil Detection on Oil Reservoir Capsule Surface

Oil reservoir capsule materials were cut into approximately 4 cm × 4 cm square pieces, washed in cyclohexane for 2 h to remove oil contamination on surface, and then dried in a vacuum drying oven at 40 °C for 4 h. After cooling, the capsule samples were ready for use. The ethanol stock solution of probe (*R*)-**1** (1.0 × 10^−3^ mol/L) was freshly prepared before each measurement. Working solutions of varying concentrations were prepared by stepwise dilution with ethanol. For visual detection, a quantified oil sample is first applied onto the capsule surface, followed immediately by the addition of the probe solution. Color changes are observed and recorded under a UV lamp. All samples were tested within 30 min.

## 4. Conclusions

A novel D–π–A solvatochromic fluorescent probe (*R*)-**1**, based on a binaphthofuran scaffold with a *p*-trifluoromethylbenzoyl moiety as acceptor, was successfully synthesized. The probe exhibits strong positive solvatochromism, with a fluorescence emission shift of 138 nm across solvents of increasing polarity, driven by an ICT mechanism. In insulating oil/ethanol systems, the probe enables high-sensitivity detection of insulating oil with an LOD of 5.88 μL/mL. On nitrile/acrylate oil reservoir capsule surfaces, the probe generates a distinct dual-color fluorescence contrast under a 365 nm UV lamp: blue-green emission from low-polarity oil stains versus orange-red emission from the high-polarity rubber/ethanol background, enabling naked-eye identification of early-stage trace oil leakage in power transformers. This method eliminates reliance on costly or non-visual techniques and provides a low-cost, rapid, on-site solution for proactive fault detection in converter transformer maintenance workflows. Future work will focus on synthesizing a series of structural analogs with systematically varied donor and acceptor strengths to establish detailed structure–solvatochromism property relationships, which may further optimize the detection sensitivity and color contrast for oil leakage inspection. Additionally, on-site validation of the probe in actual substation environments with operating transformer capsules will be carried out to evaluate its long-term stability and practical performance under real working conditions.

## Figures and Tables

**Scheme 1:**
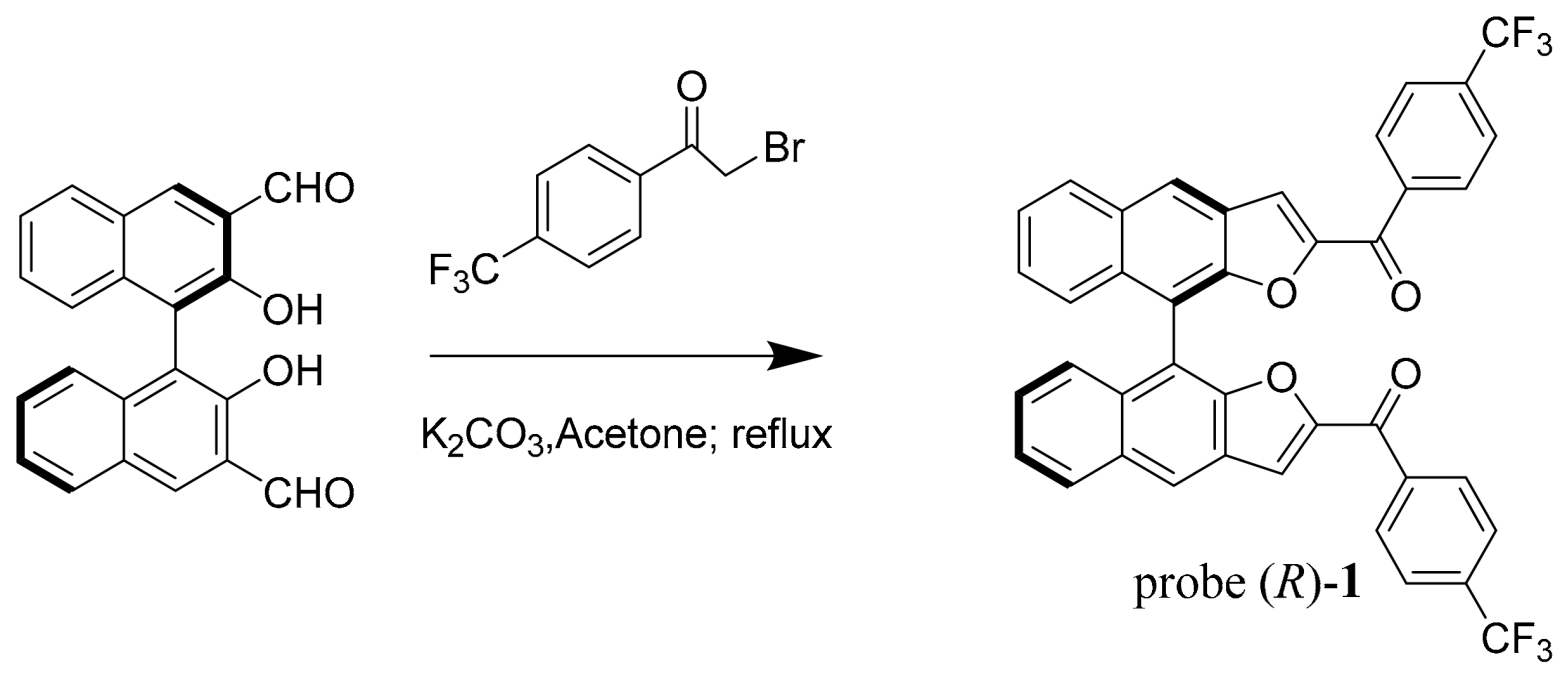
Synthesis of probe (*R*)-**1**.

**Figure 1:**
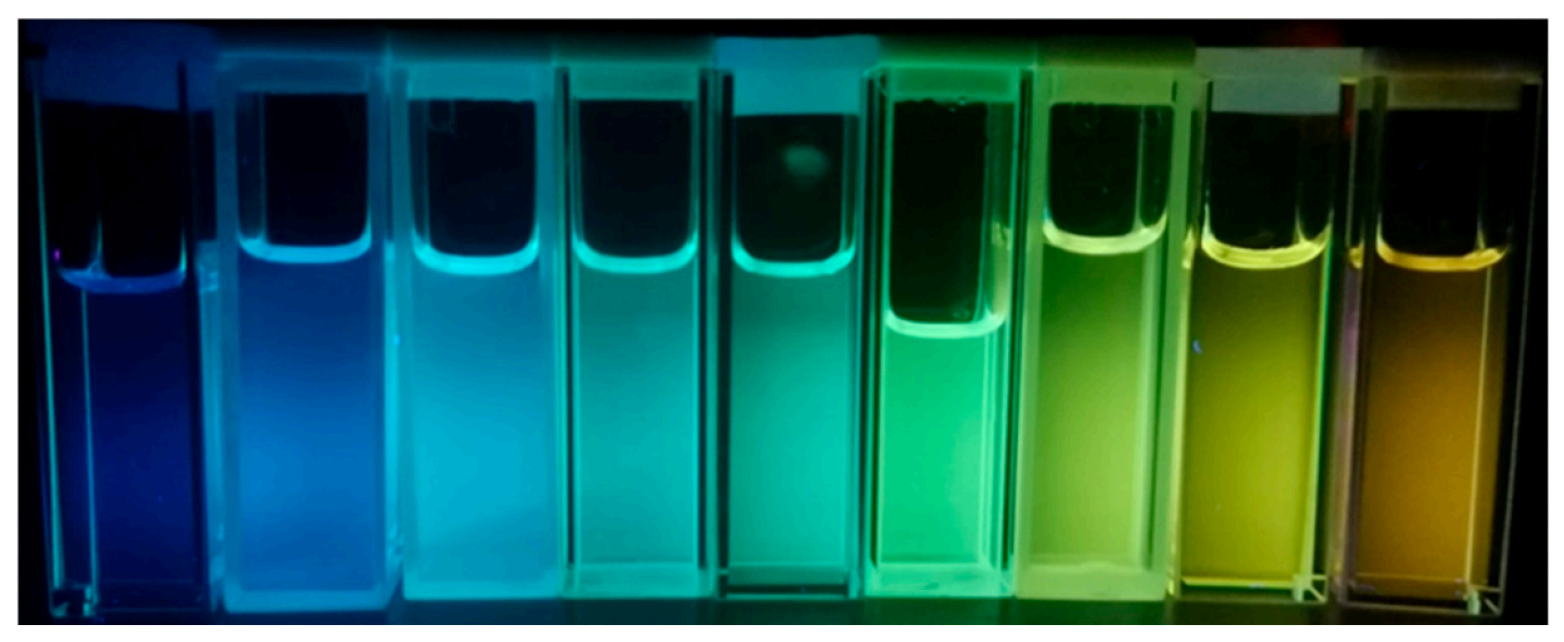
Photos of probe (*R*)-**1** in different solvents after irradiation with a 365 nm handheld UV lamp (from left to right: HEX, CCl_4_, TOL, DIO, EA, DCM, DMF, DMSO, EtOH).

**Figure 2:**
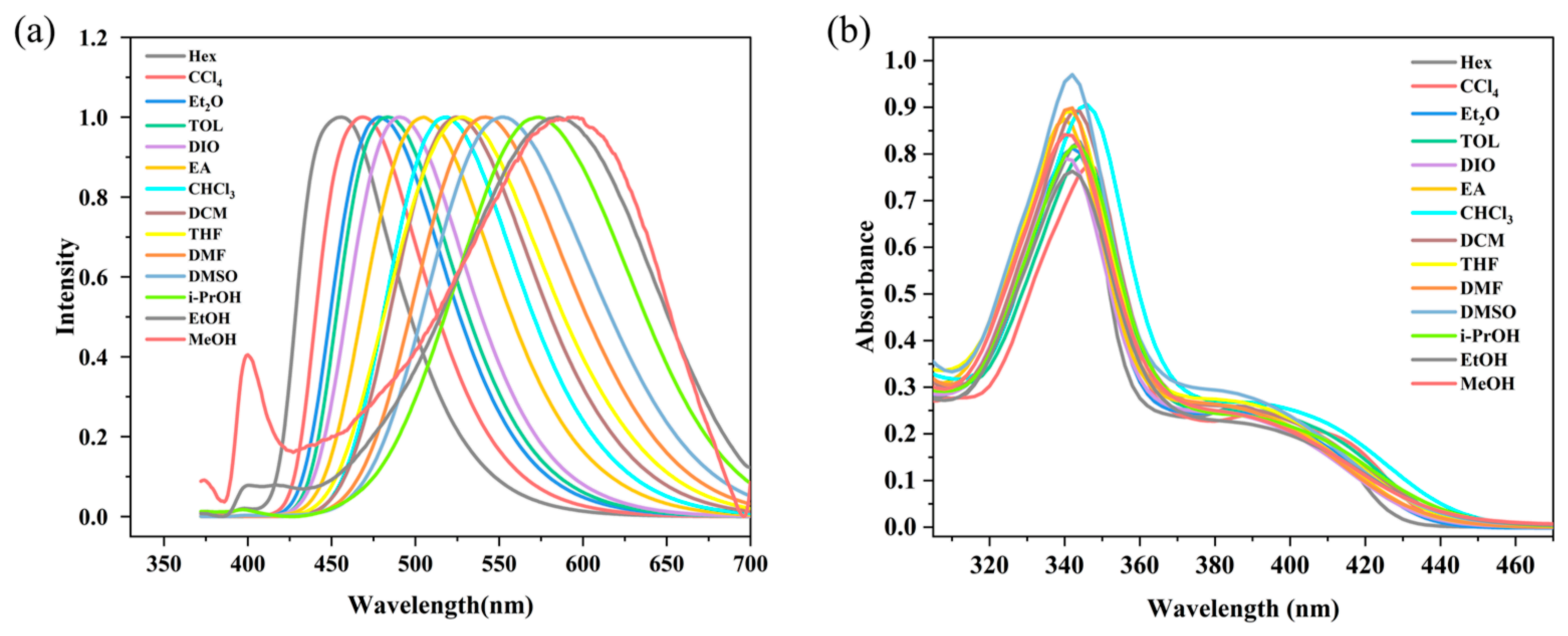
(**a**) Fluorescence emission spectra and (**b**) UV–Vis spectra of probe (*R*)-**1** (2.0 × 10^−5^ mol/L) in different solvents (λ_ex_ = 352 nm, slits: 5 nm/5 nm).

**Figure 3:**
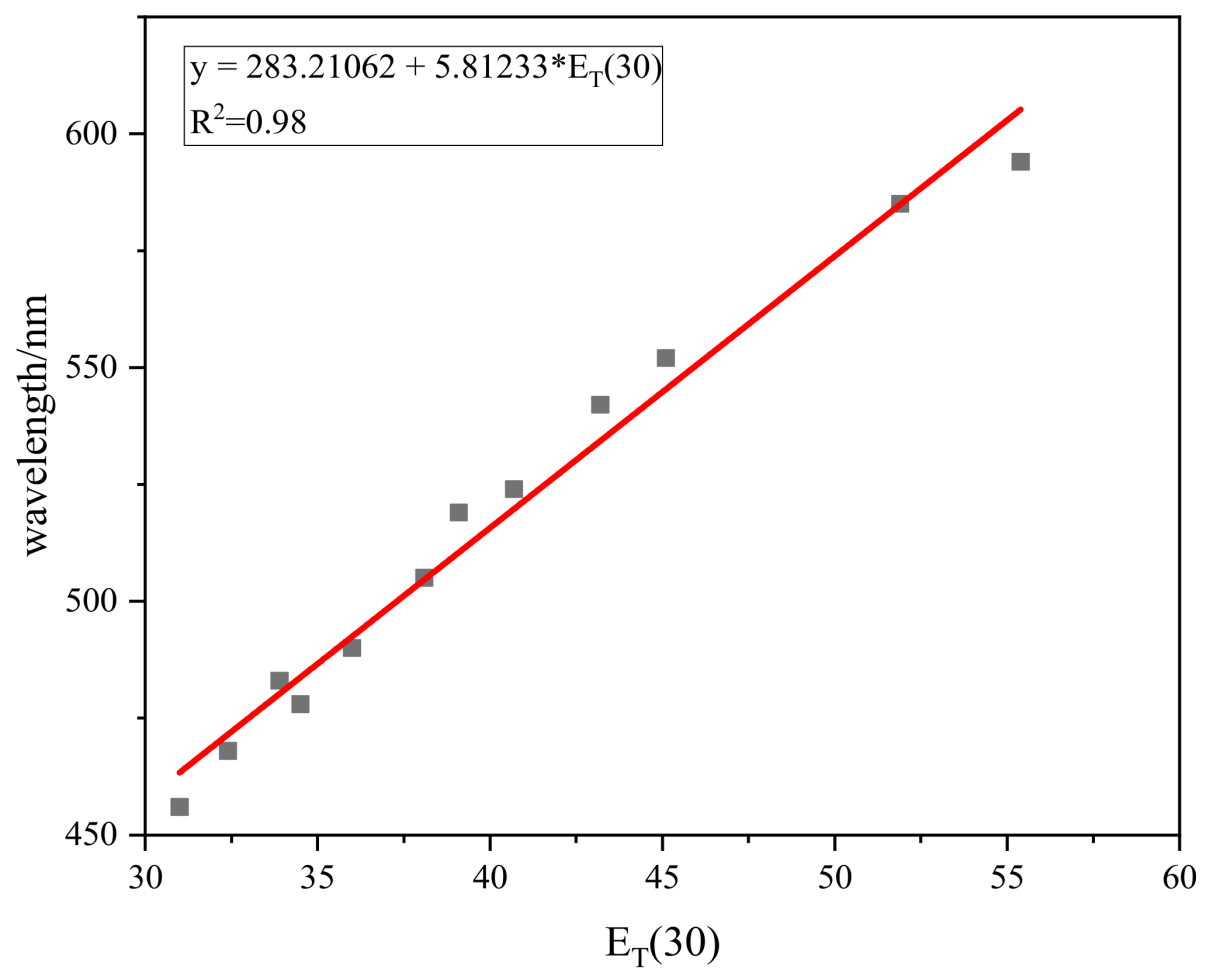
The relationship between the maximum emission wavelength (nm) of probe (*R*)-**1** and E_T_(30).

**Figure 4:**
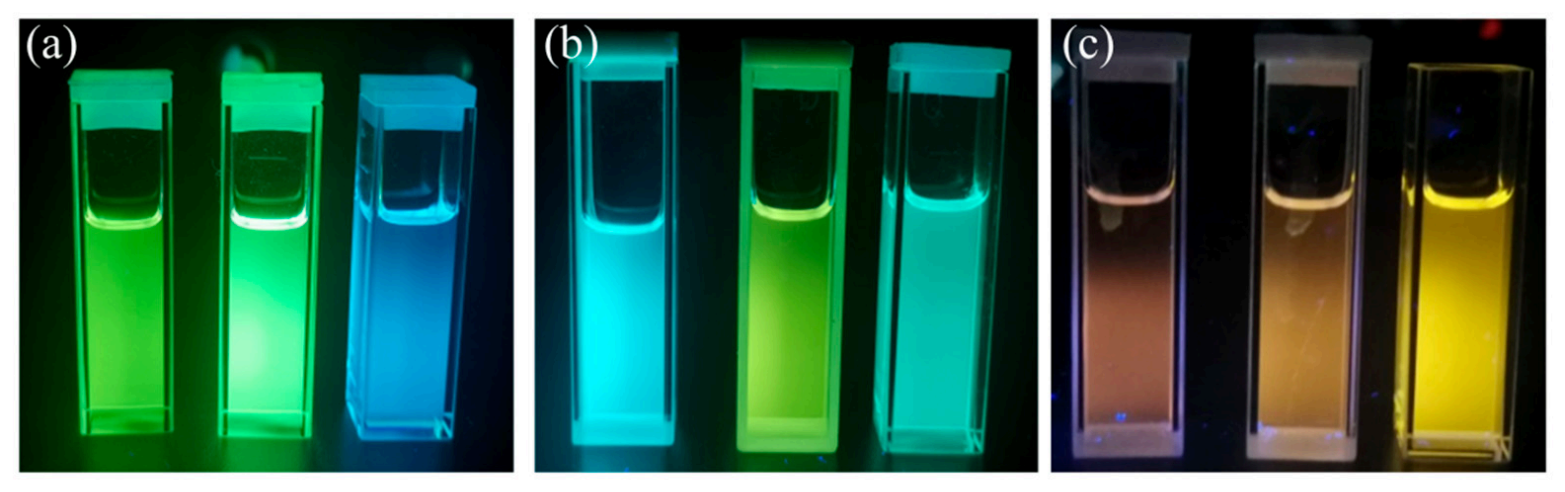
Photos of probe (*R*)-**1** in different solvents under 365 nm handheld UV lamp irradiation: (**a**) DCM, CHCl_3_, CCl_4_; (**b**) Et_2_O, THF, DIO; (**c**) MeOH, EtOH, iPrOH.

**Figure 5:**
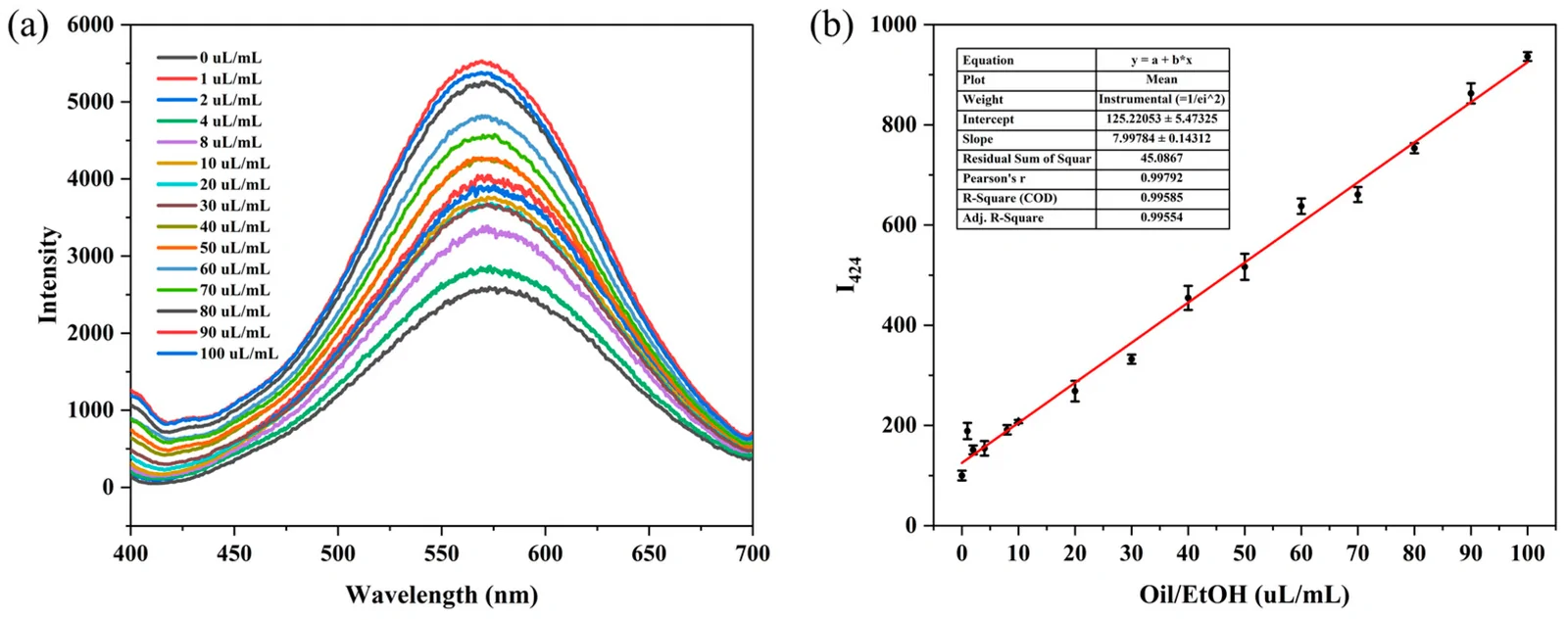
(**a**) Fluorescence titration of probe (*R*)-**1** (2 × 10^−5^ mol/L in EtOH) with 1–100 μL/mL of oil. (**b**) Linear fitting of fluorescence intensity at λ_em_ = 424 nm versus oil content in EtOH (λ_ex_ = 352 nm, slits: 5 nm/5 nm). All data points represent the mean ± standard deviation of three independent measurements (*n* = 3).

**Figure 6:**
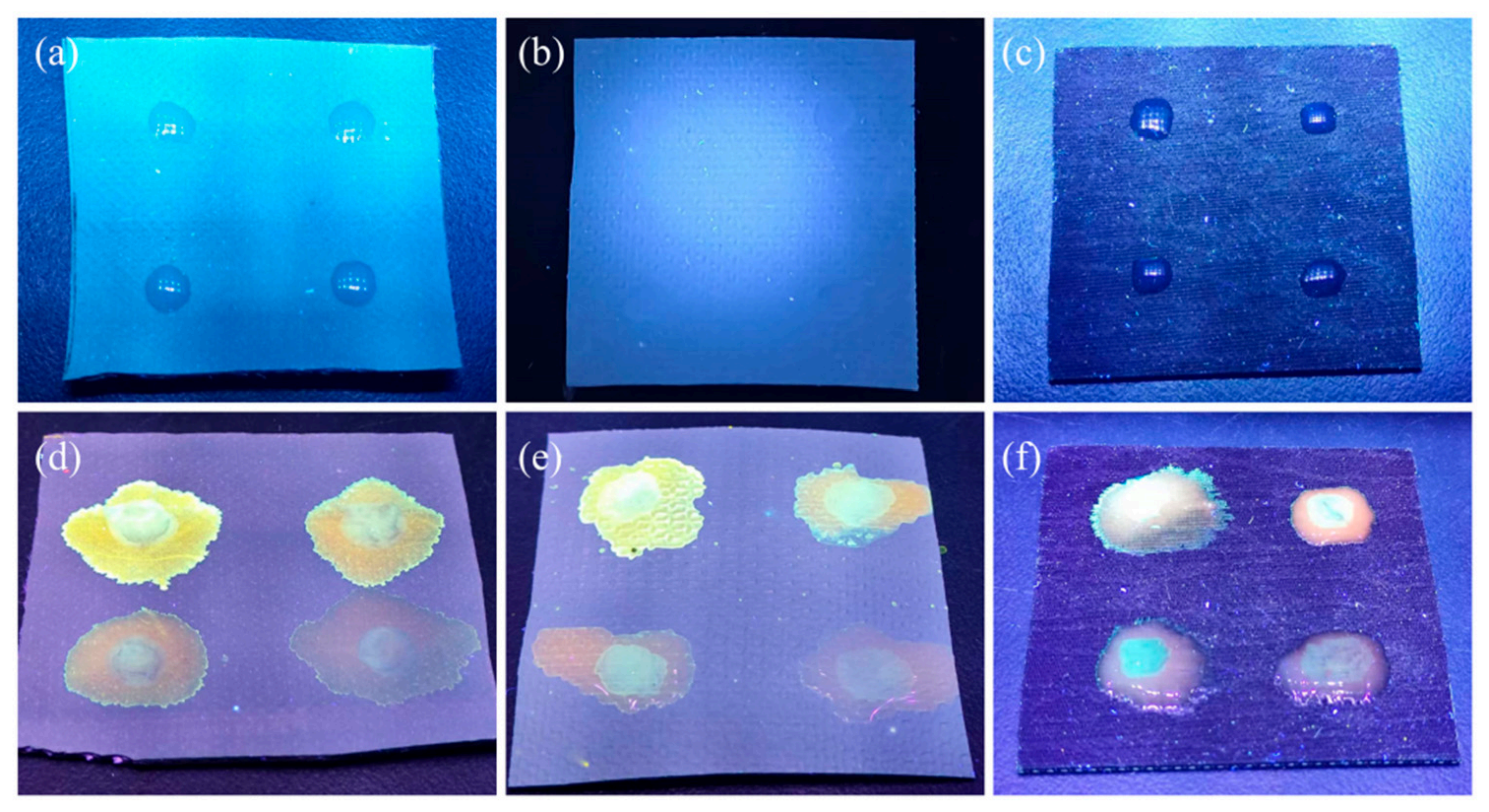
Visual detection of oil by the probe at different concentrations on three brands of capsules: (**a**–**c**) oil under UV light on capsules A, B and C in sequence (oil: 10 μL); (**d**–**f**) application of probe (*R*)-**1** on top of oil under UV light (top left: 5.0 × 10^−4^ mol/L; top right: 2.5 × 10^−4^ mol/L; bottom left: 1.0 × 10^−4^ mol/L; bottom right: 5.0 × 10^−5^ mol/L; probe solution volume: 50 μL; solvent: ethanol).

**Figure 7:**
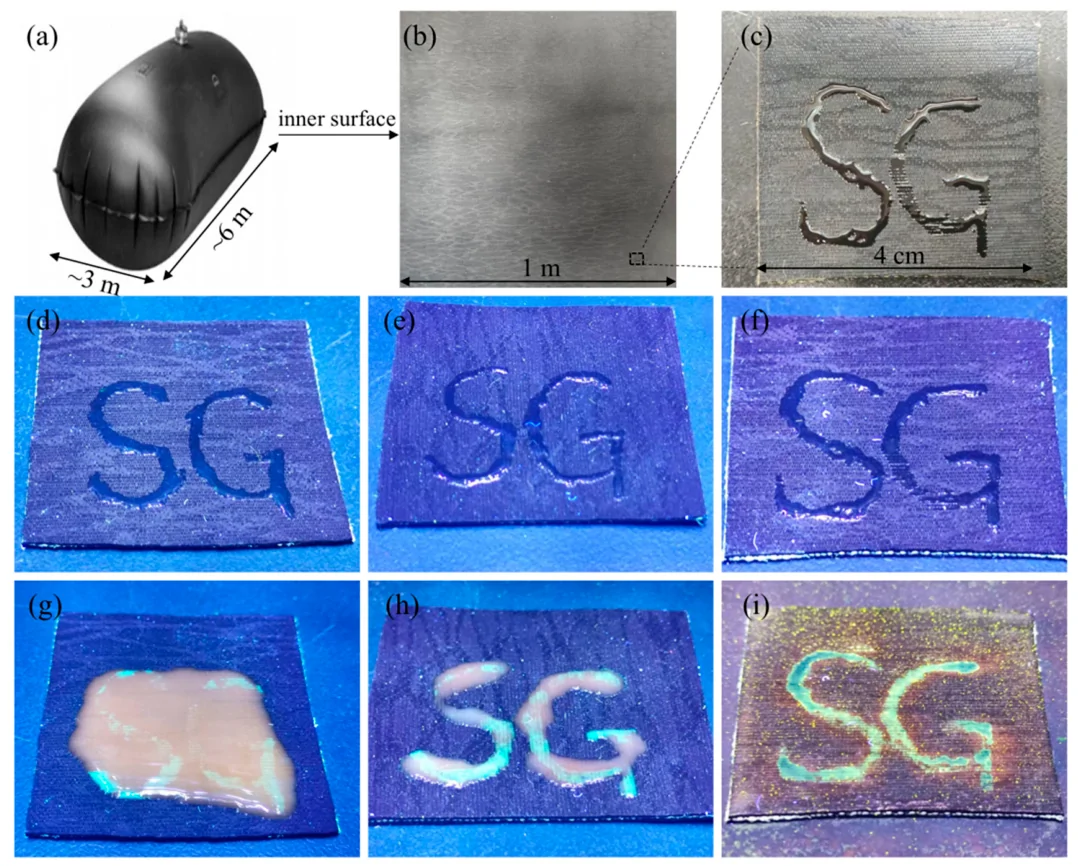
Visual detection of oil on capsule with three application methods of the probe’s ethanol solution. (**a**) Image of the oil capsules with dimensions; (**b**) the inner surface of the capsule; (**c**) capsule with oil under visual light (oil: 20 μL); (**d**–**f**) capsules with oil under UV light (oil: 20 μL); (**g**–**i**) application of probe solution under UV light by full-surface coverage, droplet deposition, and spray application in sequence (probe concentration: 1.0 × 10^−4^ mol/L in EtOH).

**Table 1:** Maximum emission wavelengths and E_T_(30) parameters of probe (*R*)-**1** in different solvents.

Solvent	Maximum Emission (nm)	E_T_ (30)	Maximum Absorption (nm)	Molar Extinction Coefficients (L·mol^−1^·cm^−1^)	Quantum Yields (%)	Stokes Shifts (nm)
HEX	456	31	344	4.13 × 10^4^	3.69	112
CCl_4_	468	32.4	346	3.88 × 10^4^	25.77	122
Et_2_O	478	34.5	342	4.06 × 10^4^	20.70	136
TOL	483	33.9	344	3.98 × 10^4^	44.11	139
DIO	490	36	340	3.95 × 10^4^	31.99	150
EA	505	38.1	340	4.37 × 10^4^	52.29	165
CHCl_3_	519	39.1	346	4.53 × 10^4^	73.06	173
DCM	524	40.7	342	4.46 × 10^4^	63.40	182
THF	528	37.4	342	4.46 × 10^4^	43.33	186
DMF	542	43.2	342	4.49 × 10^4^	37.91	200
DMSO	552	45.1	342	4.85 × 10^4^	6.95	210
iPrOH	574	-	344	4.08 × 10^4^	3.42	230
EtOH	585	51.9	342	3.82 × 10^4^	0.23	243
MeOH	594	55.4	340	4.21 × 10^4^	<0.01	254

## Data Availability

The original data presented in this study are included in the article/Supplementary Materials. Further inquiries can be directed to the corresponding author.

## Supplementary Materials

The following supporting information can be downloaded at: https://www.mdpi.com/article/10.3390/molecules31193459/s1, Figure S1: ^1^H NMR (400 MHz, Chloroform-d) of probe (*R*)-**1**; Figure S2: ^13^C NMR (101 MHz, Chloroform-d) of probe (*R*)-**1**; Figure S3: ^19^F NMR (376 MHz, Chloroform-d) of probe (*R*)-**1**; Figure S4: HRMS of probe (*R*)-**1**.
