# Supplementary material for: Ultra-Solvatochromic Probe Based on Binaphthofuran for Visual Detection of Early Trace Oil Leakage in Oil Reservoir Capsules
Source: Molecules. 2026 Sep 28;31(19):3459. doi: 10.3390/molecules31193459 (PMC13666813; doi:10.3390/molecules31193459)
Supplement: Supplementary file 1 [file molecules-31-03459-s001.zip › molecules-4539793-supplementary.pdf]

Supporting Information

Ultra-Solvatochromic Probe Based on Binaphthofuran for Visual Detection of Early Trace Oil Leakage in Oil Reservoir Capsules

Yihang Zhang [1], Hanlin Wen [1] , Fuzhi Li [1], Xinyuan Huang [1], Wenqi Fu [1], and Shanshan Yu [2,*]

[1] State Grid Sichuan Electric Power Company DC Branch, Chengdu 610021, China; zhangyh1758@sc.sgcc.com.cn (Y.Z.); wenhl0019@sc.sgcc.com.cn (H.W.); Lifz4396@sc.sgcc.com.cn (F.L.); huangxy2271@sc.sgcc.com.cn (X.H.); fuwq611X@sc.sgcc.com.cn (W.F.)

[2] Sichuan Houying Electric Power Technology Service Co., Ltd. Deyang 618000, China; yushanshan@houyinglab.cn (S.Y.)

* Correspondence: yushanshan@houyinglab.cn

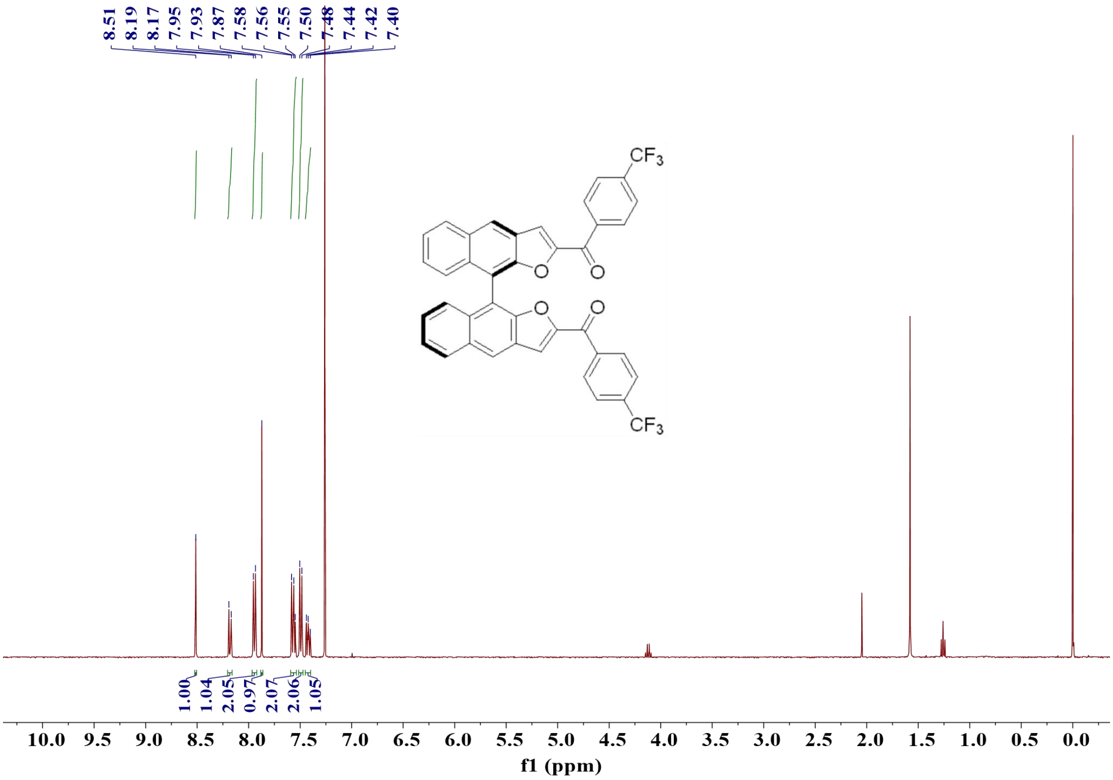

Figure S1. ^{1}H NMR (400 MHz, Chloroform-d) of probe (*R*)-**1**.

\

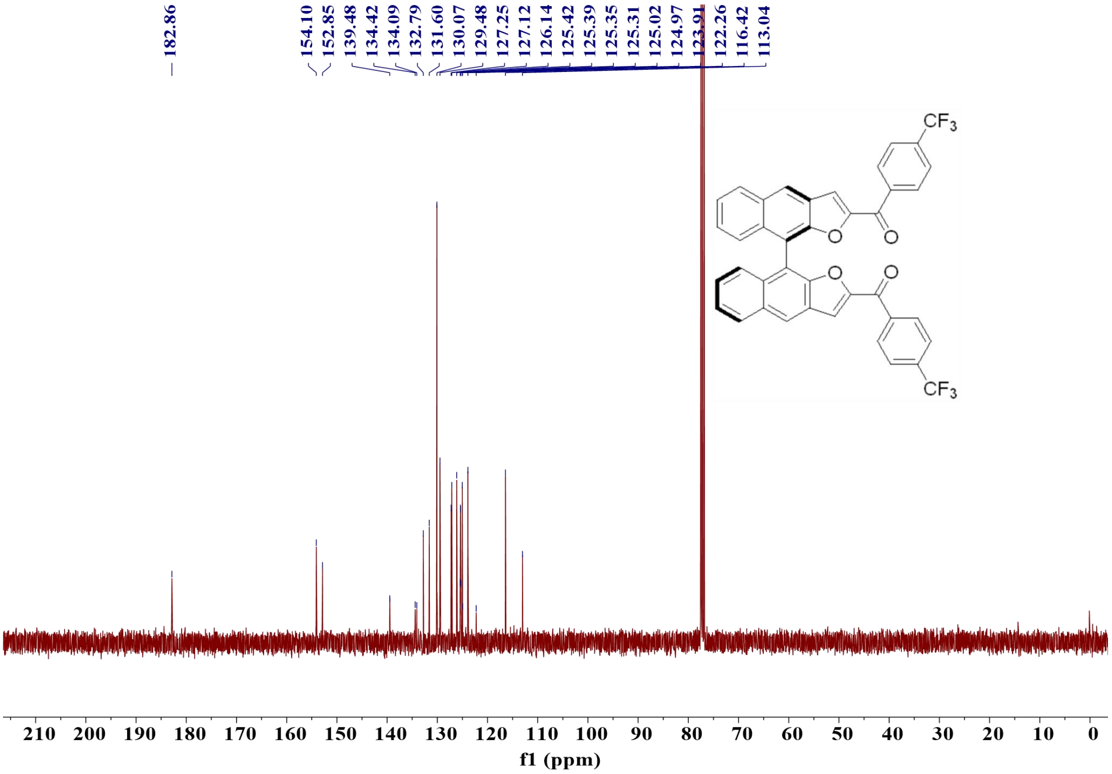

Figure S2. ^{13}C NMR (101 MHz, Chloroform-d) of probe (*R*)-**1**.

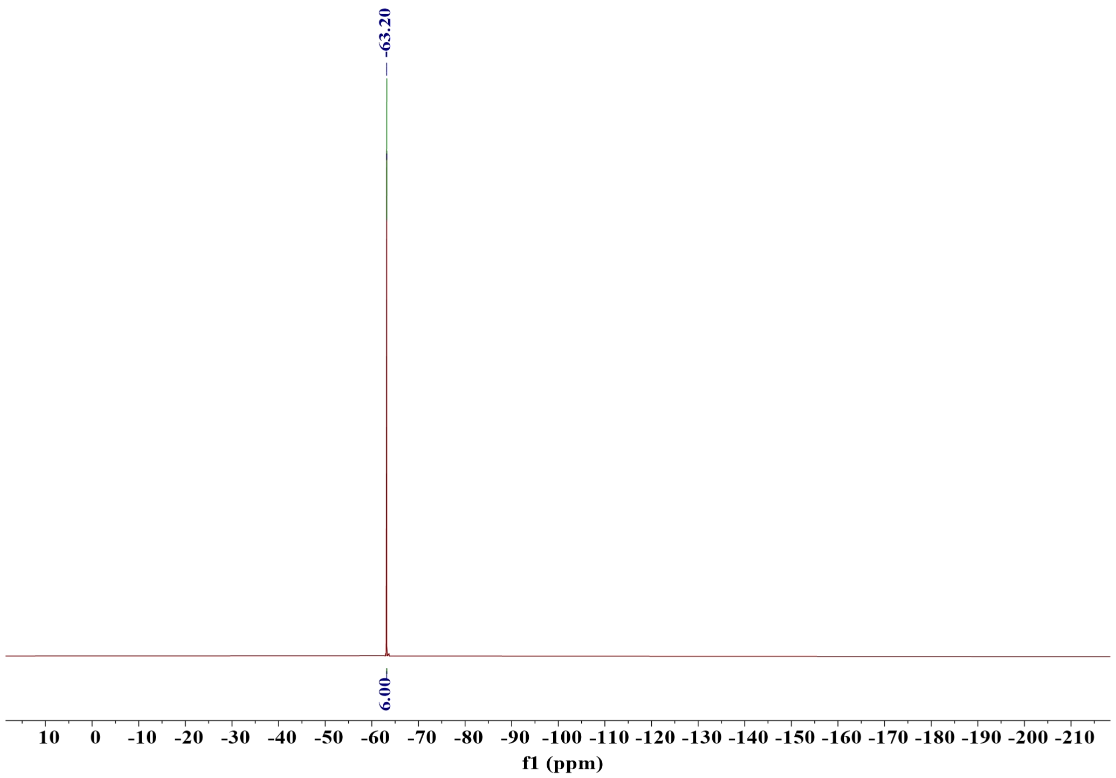

Figure S3. ^{19}F NMR (376 MHz, Chloroform-d) of probe (*R*)-**1**.

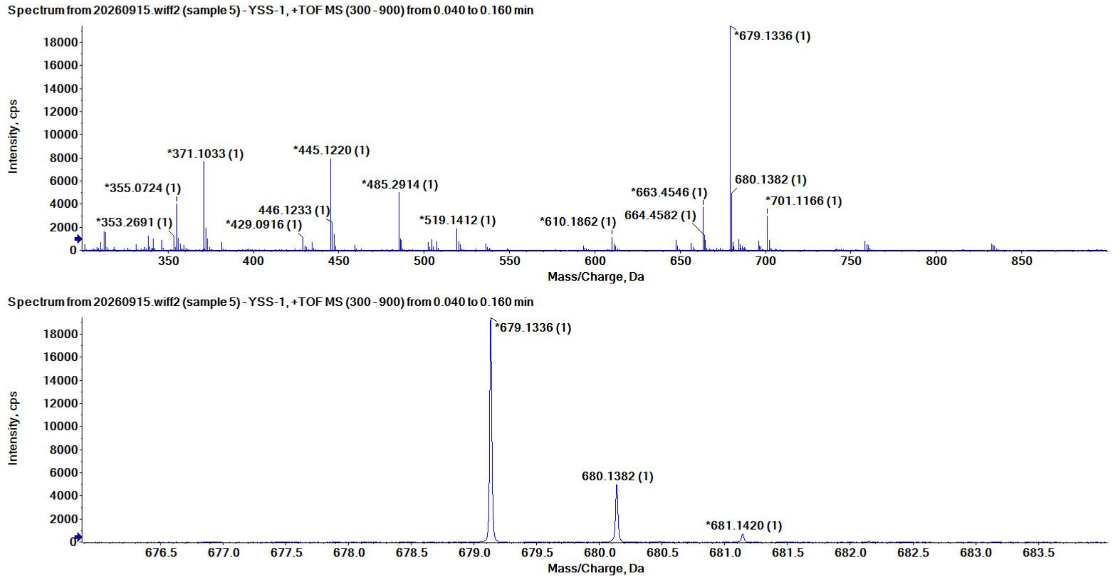

Figure S4. HRMS of probe (*R*)-**1**.
